# Perfluoroalkyl Acids in Maternal Serum and Indices of Fetal Growth: The Aarhus Birth Cohort

**DOI:** 10.1289/ehp.1510046

**Published:** 2015-10-23

**Authors:** Cathrine Carlsen Bach, Bodil Hammer Bech, Ellen Aagaard Nohr, Jørn Olsen, Niels Bjerregård Matthiesen, Eva Cecilie Bonefeld-Jørgensen, Rossana Bossi, Tine Brink Henriksen

**Affiliations:** 1 Perinatal Epidemiology Research Unit, Aarhus University Hospital, Aarhus, Denmark; 2 Horsens Regional Hospital, Horsens, Denmark; 3 Section for Epidemiology, Department of Public Health, Aarhus University, Aarhus, Denmark; 4 Research Unit for Gynecology and Obstetrics, Institute of Clinical Research, University of Southern Denmark, Odense, Denmark; 5 Department of Epidemiology, Fielding School of Public Health, University of California, Los Angeles, Los Angeles, USA; 6 Department of Pediatrics, Aarhus University Hospital, Aarhus, Denmark; 7 Centre for Arctic Health & Unit for Cellular and Molecular Toxicology, Department of Public Health, Aarhus University, Aarhus, Denmark; 8 Department of Environmental Science, Aarhus University, Roskilde, Denmark

## Abstract

**Background:**

Previous studies indicated an association between intrauterine exposure to perfluorooctane sulfonate (PFOS) or perfluorooctanoate (PFOA) and lower birth weight. However, these perfluoroalkyl acids (PFAAs) have to some extent been substituted by other compounds on which little is known.

**Objectives:**

We investigated the association between specific PFAAs and birth weight, birth length, and head circumference at birth.

**Methods:**

We studied 1,507 mothers and their children from the Aarhus Birth Cohort (2008–2013). Nulliparous women were included during pregnancy, and serum levels of 16 PFAAs were measured between 9 and 20 completed gestational weeks (96% within 13 weeks). For compounds with quantifiable values in > 50% of samples (7 compounds), we report the associations with birth weight, birth length, and head circumference at birth determined by multivariable linear regression.

**Results:**

Estimated mean birth weights were lower among women with serum perfluorohexane sulfonate, perfluoroheptane sulfonate, and PFOS concentrations above the lowest exposure quartile, but we found no consistent monotonic dose–response patterns. These associations were stronger when the population was restricted to term births (n = 1,426). For PFOS, the birth weight estimates for the highest versus lowest quartile were –50 g (95% CI: –123, 23 g) in all births and –62 g (95% CI: –126, 3 g) in term births. For the other PFAAs, the direction of the associations was inconsistent, and no overall association with birth weight was apparent. No PFAAs were associated with birth length or head circumference at birth.

**Conclusions:**

Overall, we did not find strong or consistent associations between PFAAs and birth weight or other indices of fetal growth, though estimated mean birth weights were lower among those with exposures above the lowest quartile for some compounds.

**Citation:**

Bach CC, Bech BH, Nohr EA, Olsen J, Matthiesen NB, Bonefeld-Jørgensen EC, Bossi R, Henriksen TB. 2016. Perfluoroalkyl acids in maternal serum and indices of fetal growth: the Aarhus Birth Cohort. Environ Health Perspect 124:848–854; http://dx.doi.org/10.1289/ehp.1510046

## Introduction

Perfluoroalkyl acids (PFAAs) are human-made chemicals found in various products, for example, food items and packaging, pots and pans, and textiles such as carpets, furniture, shoes, and clothing (Kantiani et al. 2010). Specific PFAAs [perfluorooctane sulfonate (PFOS) and perfluorooctanoic acid (PFOA)] have been voluntarily phased out or regulated in some parts of the world (Environment Canada 2010; European Parliament 2006; UNEP 2009; U.S. EPA 2000, 2006), and these compounds have to some extent been replaced by novel, similar compounds.

PFAAs accumulate in the human body (Butenhoff et al. 2006) and are persistent in the environment. Some have been shown to cross the placenta (Fei et al. 2007; Inoue et al. 2004; Kim et al. 2011; Midasch et al. 2007), and it has been hypothesized that they may affect fetal growth and development, possibly due to interaction with the peroxisome proliferator-activated receptor alpha (Abbott 2009), estrogen biosynthesis or interaction with receptors *in vitro* (Benninghoff et al. 2011; Du et al. 2013; Henry and Fair 2011; Kjeldsen and Bonefeld-Jørgensen 2013), thyroid hormone signaling (Du et al. 2013; Wang et al. 2013; Thibodeaux et al. 2003; Lau et al. 2003; Long et al. 2013; Inoue et al. 2004; Kim et al. 2011), or lipid metabolism (Apelberg et al. 2007; Thibodeaux et al. 2003). It is also plausible that exposures could affect the mother’s appetite and food intake, or have direct effects on placental growth and/or function.

In animals, several studies have found lower birth weight with exposure to PFOS (Luebker et al. 2005; Thibodeaux et al. 2003), PFOA (Koustas et al. 2014), and perfluoroundecanoic acid (PFUnA) (Takahashi et al. 2014). However, exposure levels were higher than average environmental exposures in humans. Epidemiological studies have investigated the association between exposure to PFOS and PFOA and birth weight or related outcomes (Apelberg et al. 2007; Arbuckle et al. 2013; Chen et al. 2012; Darrow et al. 2013; Fei et al. 2007; Hamm et al. 2010; Inoue et al. 2004; Kishi et al. 2015; Lee et al. 2013; Maisonet et al. 2012; Monroy et al. 2008; Robledo et al. 2015; Stein et al. 2009; Washino et al. 2009; Whitworth et al. 2012b; Wu et al. 2012). Even though most studies indicated an association with lower birth weight, in particular for PFOA, many studies had limited power and thus low precision due to small sample sizes (Bach et al. 2015). Few small studies (all with < 500 participants) investigated other PFAAs such as perfluorononanoic acid [PFNA (Arbuckle et al. 2013; Chen et al. 2012; Monroy et al. 2008; Robledo et al. 2015)], PFUnA (Chen et al. 2012), perfluorohexane sulfonate [PFHxS (Arbuckle et al. 2013; Hamm et al. 2010; Lee et al. 2013; Maisonet et al. 2012; Monroy et al. 2008)], perfluorodecanoic acid [PFDA (Robledo et al. 2015)], and perfluorooctane sulfonamide (Robledo et al. 2015) and found no consistent associations. To our knowledge, the present study is the largest to address PFAAs other than PFOS and PFOA to date. We aimed to examine the association between maternal serum levels of several PFAAs and birth weight, birth length, and head circumference at birth in a large sample of pregnant women exposed to present (2008–2013) population levels of PFAAs. Additionally, we aimed to investigate the association between PFAAs and gestational age at birth and preterm birth.

## Methods

### The Aarhus Birth Cohort and Biobank

Annually, approximately 4,500 women give birth at the Department of Obstetrics and Gynecology, Aarhus University Hospital, Denmark. The ongoing Aarhus Birth Cohort (ABC) was established in 1989 and contains self-administered questionnaire data from pregnant women who plan to give birth at the hospital. Most women complete the questionnaire at approximately 12 weeks of gestation. The cohort also contains information about birth outcomes collected by clinical staff (attending midwives) immediately after delivery using a structured birth registration form. Trained research midwives validated birth data until January 2013, and after this date the authors reviewed the registry records. So far, the cohort contains information on > 100,000 women (Larsen et al. 2013).

A biobank was added to the cohort in 2008 and contains blood samples from pregnant women, their partners, and umbilical cord material (Mortensen et al. 2013). Most maternal blood samples were obtained at approximately gestational week 12. All samples were processed within 2 hr and stored at –80°C until further analysis. All participants provided written informed consent accepting that blood samples are stored in a biobank, that the questionnaire data are stored electronically, and that all information can be used for future research approved by the proper authorities (Mortensen et al. 2013). Currently, the biobank contains blood samples from > 10,000 women.

### Selection of Participants

For this study, women in the cohort were eligible if they donated a blood sample between 9 and 20 completed gestational weeks, gave birth to a live-born, singleton neonate, and were nulliparous. From the women who fulfilled these criteria (*n* = 2,853) we randomly sampled 1,533 women recruited during 2008–2013 (220–290 participants annually). Of these women, 1,507 (98%) had complete data on exposure, birth weight, and covariates and were included in the final study population. Regarding birth length, 1,499 (98%) participants were included, and for head circumference, 1,494 (97%) participants were included in the analyses. Approval was obtained from the Danish Data Protection Agency (reference 2012-41-1288), and the data collection was accepted by the Danish National Committee on Health Research Ethics (reference M-20110054).

### Exposure Assessment

We measured the levels of 16 PFAAs in maternal serum and included compounds where at least 50% of the samples were above the lower limit of quantification (LOQ) (Table 1). PFAA analysis was performed at the Department of Environmental Science, Aarhus University, by use of high performance liquid chromatography–tandem mass spectrometry after solid phase extraction (Liew et al. 2014).

**Table 1:** Perfluoroalkyl acid abbreviations, limits of quantification (LOQ), levels above the LOQ, and exposure distributions for included compounds measured in 1,507 serum samples from the Aarhus Birth Cohort, 2008–2013.

Full name of PFAA	Abbreviation	LOQ	% above LOQ	Median (IQR)	Quartile 1	Quartile 2	Quartile 3	Quartile 4
Perfluorohexane sulfonate	PFHxS	0.08	99.9	0.5 (0.4–0.6)	< LOQ–0.36	0.37–0.47	0.48–0.63	0.64–6.82
Perfluoroheptane sulfonate	PFHpS	0.11	76	0.2 (0.1–0.2)	< LOQ–0.11	0.12–0.16	0.17–0.21	0.22–1.16
Perfluorooctane sulfonate	PFOS	0.28	99.9	8.3 (6.0–10.8)	< LOQ–6.02	6.03–8.29	8.30–10.80	10.81–36.10
Perfluorooctanoic acid	PFOA	0.20	99.9	2.0 (1.5–2.6)	< LOQ–1.53	1.54–2.02	2.03–2.64	2.65–15.10
Perfluorononanoic acid	PFNA	0.27	99	0.8 (0.6–1.0)	< LOQ–0.60	0.61–0.75	0.76–0.97	0.98–4.69
Perfluorodecanoic acid	PFDA	0.09	99	0.3 (0.2–0.4)	< LOQ–0.24	0.25–0.31	0.32–0.42	0.43–2.87
Perfluoroundecanoic acid	PFUnA	0.15	87	0.3 (0.2–0.4)	< LOQ–0.20	0.21–0.29	0.30–0.44	0.44–2.47
Abbreviations: IQR, Interquartile range; PFAA, perfluoroalkyl acid. All concentrations are in nanograms per milliliter.								

### Outcomes

The outcomes available in the cohort included birth weight (continuous and *z*-score), birth length, and head circumference at birth. *z*-Scores were calculated by standardization of birth weight for gestational age according to the most recent (1996) Scandinavian fetal reference (Marsál et al. 1996). Furthermore, we studied the gestational age at birth and the odds for preterm birth (birth before 37 weeks and 0 days of gestation). Gestational age at birth was determined by first trimester ultrasound measurements. We identified no implausible values of gestational age at birth (< 24 weeks or > 45 weeks). We identified birth weight and gestational age mismatches (Alexander et al. 1996), and participants with implausible combinations (*n* = 5) were excluded from the analyses of birth weight along with six participants with missing birth weight.

### Statistical Analysis

We performed multivariable linear regression with robust standard errors (Huber–White sandwich estimator) to estimate the association between individual PFAAs and continuous birth weight and birth weight *z*-scores, birth length, head circumference at birth, and gestational age at birth. The association between levels of PFAAs and preterm birth was analyzed by logistic regression. We substituted PFAA values below the LOQ with the LOQ divided by the square root of 2. Levels of PFAAs were divided into quartiles with the lowest category used as reference. Moreover, we assessed the associations between continuous exposure measures and the above-mentioned outcomes. The continuous exposure measures were rescaled to assess the change in outcomes with a 0.1-ng/mL increase in PFAA levels because most of the compounds had median values < 1 ng/mL. To enhance comparability of individual PFAAs within our study, we also modeled estimates per interquartile range of exposure. In addition, PFAA levels were modeled by the use of restricted cubic splines with prespecified knots according to the quartile boundaries for each PFAA.

We identified covariates to include in the analyses by directed acyclic graphs (DAGs) (see Figures S1 and S2). The analyses were adjusted for maternal age (continuous), maternal prepregnancy body mass index [BMI (continuous)], and maternal level of education (four categories). Furthermore we conditioned on parity by restricting to nulliparous women. Information on maternal age and parity was available in the birth registration form, and information on BMI and maternal level of education was extracted from the questionnaire. There were no missing values for maternal age. Few values were missing for BMI (*n* = 11) and level of education (*n* = 4).

In a secondary analysis, we included gestational age (continuous) in the birth weight model in addition to the other covariates. We did this to compare our findings with the existing literature, even though we are aware of the caveats of adjustment for a potential intermediate (Wilcox et al. 2011). These considerations apply to birth weight *z*-scores as well. We also restricted the analyses to children born at term (≥ 37 weeks of gestation). Due to previous reports on sex differences concerning the investigated association (Andersen et al. 2010; Maisonet et al. 2012; Robledo et al. 2015; Washino et al. 2009), we performed the analyses of birth weight separate for each sex, as well as pooled. We examined the importance of the gestational age at blood draw by restriction to participants who gave a blood sample within 13 completed gestational weeks [*n* = 1,440 (96%)]. STATA statistical software version 12 (StataCorp, College Station, TX, USA) was used for all the statistical analyses.

## Results

For seven of the measured PFAAs, more than 50% of the samples had concentrations above the lower limit of quantification [PFHxS, perfluoroheptane sulfonate (PFHpS), PFOS, PFOA, PFNA, PFDA, and PFUnA]. LOQ, levels above the LOQ, and distributions of the included PFAAs are listed in Table 1, and corresponding information regarding the excluded compounds is listed in Table S1. Pearson’s correlations for the seven included PFAAs are shown in Table S2. The two PFAAs with the highest correlation were PFNA and PFDA (Pearson’s correlation = 0.85), and the lowest correlation was found for PFUnA and PFHxS (Pearson’s correlation = 0.14).

Women with exposure levels in the higher quartiles were slightly older than those with exposure levels in the lower quartiles—the median ages were 29 years in the lowest quartiles and 30–31 years in the highest (Table 2). Maternal prepregnancy BMI did not differ according to exposure levels. For women with the three higher levels of education, there was not much difference between exposure distributions, but for the group of women with the lowest educational level, 38–50% of women had exposure levels in the lowest PFAA quartiles.

**Table 2:** Maternal characteristics according to early pregnancy levels of perfluoroalkyl acids in 1,507 mothers from the Aarhus Birth Cohort, 2008–2013.

PFAA	Age (years) [median (IQR)]	Prepregnancy BMI (kg/m^2^) [median (IQR)]	Education (%)			
			1 (*n* = 42)	2 (*n* = 439)	3 (*n* = 580)	4 (*n* = 446)
PFHxS
Q1	29 (27–31)	22 (20–25)	50	27	27	19
Q2	29 (27–31)	22 (21–25)	19	24	26	25
Q3	30 (27–32)	22 (21–25)	21	27	23	25
Q4	31 (29–33)	22 (20–25)	10	23	24	31
PFHpS
Q1	29 (27–32)	22 (20–24)	48	23	26	24
Q2	29 (27–32)	22 (20–24)	14	28	25	26
Q3	29 (27–32)	22 (20–25)	21	24	26	22
Q4	30 (28–33)	22 (21–25)	17	24	22	28
PFOS
Q1	29 (27–32)	22 (20–25)	45	25	27	22
Q2	29 (27–32)	22 (20–25)	19	25	25	24
Q3	29 (27–32)	22 (20–24)	22	27	23	29
Q4	30 (28–32)	22 (21–25)	14	23	25	25
PFOA
Q1	29 (27–32)	22 (21–26)	43	23	27	23
Q2	29 (27–32)	22 (20–24)	28	25	26	26
Q3	29 (27–31)	22 (20–25)	10	27	23	25
Q4	30 (28–32)	22 (21–25)	19	25	24	26
PFNA
Q1	29 (27–32)	22 (20–25)	48	26	25	21
Q2	30 (28–32)	22 (21–25)	17	26	25	27
Q3	30 (27–32)	22 (20–24)	10	24	24	27
Q4	30 (28–32)	22 (21–25)	26	24	26	24
PFDA
Q1	29 (27–32)	22 (20–25)	38	27	27	20
Q2	29 (27–31)	22 (21–25)	19	25	26	24
Q3	30 (27–33)	22 (20–25)	10	25	23	29
Q4	30 (28–32)	22 (20–24)	33	23	24	28
PFUnA
Q1	29 (27–32)	22 (21–26)	48	30	27	18
Q2	29 (27–32)	22 (20–25)	19	25	26	22
Q3	30 (27–32)	22 (20–25)	17	23	23	31
Q4	30 (28–32)	22 (20–24)	17	22	24	29
Abbreviations: IQR, interquartile range; PFAA, perfluoroalkyl acid; Q, quartile. For specific PFAA abbreviations see Table 1. Definitions of highest completed maternal education: 1. Municipal primary and lower secondary school; 2. upper secondary school, or 1–2 years of vocational training; 3. additional 3–4 years of education, e.g., Bachelor’s degree; 4. > 4 additional years of education, e.g., Master’s degree.						

For three perfluoroalkane sulfonic acids (PFHxS, PFHpS, and PFOS) estimated mean birth weights were lower for all quartiles above the first quartile (Table 3). Only PFHxS showed some indication of a monotonic dose–response relationship with birth weight. Further adjustment for gestational age did not change the results in any consistent direction, but the associations for PFHxS, PFHps, and PFOS were stronger in term births (*n* = 1,426). Continuous exposure measures of these compounds were not consistently associated with lower estimated mean birth weights.

**Table 3:** Maternal levels of perfluoroalkyl acids and birth weight in 1,507 children from the Aarhus Birth Cohort, 2008–2013.

PFAA/exposure scale	Birth weight (g)					Birth weight *z*-score	
	Mean ± SD	Crude	Adjusted^*a*^ (95% CI)	Adjusted^*b*^ (95% CI)	Restricted^*c*^ (95% CI)	Crude	Adjusted^*a*^ (95% CI)
PFHxS
Q1	3,460 ± 556	Reference	Reference	Reference	Reference	Reference	Reference
Q2	3,452 ± 460	–9	–15 (–87, 57)	–11 (–67, 44)	–41 (–105, 24)	0.00	–0.01 (–0.15, 0.12)
Q3	3,436 ± 504	–24	–25 (–100, 50)	–21 (–79, 37)	–34 (–101, 32)	–0.04	–0.03 (–0.17, 0.11)
Q4	3,424 ± 511	–37	–29 (–106, 47)	–49 (–109, 11)	–49 (–118, 19)	–0.13	–0.11 (–0.25, 0.03)
Per IQR (0.3 ng/mL)		–9	–7 (–28, 14)	–13 (–30, 3)	–11 (–32, 9)	–0.04	–0.03 (–0.07, 0.01)
Per 0.1 ng/mL		–3	–2 (–10, 5)	–5 (–11, 1)	–4 (–12, 3)	–0.01	–0.01 (–0.03, 0.00)
PFHpS
Q1	3,473 ± 509	Reference	Reference	Reference	Reference	Reference	Reference
Q2	3,418 ± 540	–56	–52 (–126, 21)	–20 (–75, 35)	–46 (–109, 17)	–0.05	–0.04 (–0.17, 0.10)
Q3	3,446 ± 478	–27	–34 (–105, 36)	–50 (–107, 6)	–56 (–118, 7)	–0.10	–0.11 (–0.24, 0.02)
Q4	3,436 ± 504	–38	–42 (–115, 31)	–43 (–100, 14)	–63 (–129, 2)	–0.10	–0.10 (–0.23, 0.04)
Per IQR (0.1 ng/mL)		–8	–12 (–40, 17)	–17 (–40, 6)	–23 (–50, 4)	–0.03	–0.04 (–0.09, 0.02)
Per 0.1 ng/mL		–7	–11 (–37, 16)	–15 (–36, 5)	–21 (–45, 3)	–0.03	–0.04 (–0.09, 0.02)
PFOS
Q1	3,481 ± 520	Reference	Reference	Reference	Reference	Reference	Reference
Q2	3,397 ± 518	–84	–86 (–159, –13)	–66 (–122, –11)	–93 (–157, –29)	–0.15	–0.15 (–0.29, –0.02)
Q3	3,461 ± 486	–20	–21 (–91, 48)	–30 (–86, 26)	–50 (–113, 13)	–0.07	–0.06 (–0.19, 0.07)
Q4	3,431 ± 510	–50	–50 (–123, 23)	–58 (–105, 8)	–62 (–126, 3)	–0.11	–0.11 (–0.25, 0.02)
Per IQR (4.8 ng/mL)		–1	–2 (–30, 26)	–8 (–30, 14)	–14 (–40, 11)	–0.02	–0.02 (–0.07, 0.04)
Per 0.1 ng/mL		0	0 (–1, 1)	0 (–1.0)	0 (–1, 0)	0.00	0.00 (0.00, 0.00)
PFOA
Q1	3,441 ± 536	Reference	Reference	Reference	Reference	Reference	Reference
Q2	3,419 ± 522	–22	–1 (–75, 74)	3 (–54, 59)	–36 (–101, 30)	–0.04	0.009 (–0.13, 0.14)
Q3	3,458 ± 488	17	28 (–45, 102)	15 (–42, 72)	–7 (–73, 58)	0.01	0.04 (–0.09, 0.17)
Q4	3,455 ± 488	13	26 (–47, 98)	9 (–47, 64)	4 (–59, 67)	–0.005	0.03 (–0.10, 0.16)
Per IQR (1.1 ng/mL)		19	21 (–1, 44)	7 (–10, 23)	13 (–6, 33)	0.01	0.02 (–0.02, 0.06)
Per 0.1 ng/mL		2	2 (–1, 4)	1 (–1, 2)	1 (–1, 3)	0.00	0.00 (0.00, 0.00)
PFNA
Q1	3,458 ± 544	Reference	Reference	Reference	Reference	Reference	Reference
Q2	3,452 ± 496	–6	–12 (–85, 61)	–20 (–77, 37)	–26 (–92, 40)	–0.02	–0.03 (–0.17, 0.10)
Q3	3,391 ± 501	–67	–63 (–137, 11)	–43 (–100, 14)	–72 (–137, –6)	–0.10	–0.08 (–0.22, 0.05)
Q4	3,471 ± 490	14	11 (–62, 84)	3 (–54, 61)	10 (–55, 75)	0.03	0.02 (–0.11, 0.16)
Per IQR (0.4 ng/mL)		16	15 (–7, 38)	8 (–9, 25)	11 (–9, 31)	0.02	0.02 (–0.02, 0.06)
Per 0.1 ng/mL		4	4 (–2, 10)	2 (–2, 7)	3 (–2, 9)	0.01	0.01 (0.00, 0.02)
PFDA
Q1	3,461 ± 554	Reference	Reference	Reference	Reference	Reference	Reference
Q2	3,432 ± 492	–29	–34 (–107, 39)	–45 (–103, 13)	–55 (–120, 11)	–0.09	–0.10 (–0.24, 0.04)
Q3	3,411 ± 506	–50	–44 (–119, 31)	–55(–113, 2)	–52 (–117, 13)	–0.15	–0.13 (–0.26, 0.01)
Q4	3,468 ± 480	7	17 (–56, 90)	2 (–55, 59)	4 (–60, 69)	–0.01	0.02 (–0.12, 0.15)
Per IQR (0.2 ng/mL)		11	15 (–6, 37)	9 (–7, 24)	13 (–6, 31)	0.01	0.02 (–0.01, 0.06)
Per 0.1 ng/mL		6	9 (–3, 21)	5 (–4, 13)	7 (–3, 17)	0.01	0.01 (–0.01, 0.03)
PFUnA
Q1	3,449 ± 532	Reference	Reference	Reference	Reference	Reference	Reference
Q2	3,439 ± 510	–10	3 (–71, 76)	–3 (–61, 55)	4 (–60, 69)	–0.04	–0.007 (–0.15, 0.13)
Q3	3,459 ± 513	10	29 (–46, 105)	17 (–42, 76)	24 (–43, 91)	–0.02	0.03 (–0.11, 0.18)
Q4	3,425 ± 480	–24	8 (–65, 81)	–13 (–72, 45)	–15 (–81, 51)	–0.11	–0.03 (–0.17, 0.11)
Per IQR (0.2 ng/mL)		–9	0 (–21, 21)	–5 (–22, 11)	–8 (–27, 11)	–0.03	–0.01 (–0.05, 0.03)
Per 0.1 ng/mL		–4	0 (–8, 9)	–2 (–9, 5)	–3 (–11, 5)	–0.01	0.00 (–0.02, 0.01)
Abbreviations: IQR, interquartile range; Q, quartile. For PFAA abbreviations see Table 1. ^***a***^Adjusted for maternal age, prepregnancy BMI and educational level. ^***b***^Additionally adjusted for gestational age (continuous). ^***c***^Adjusted as model A and restricted to term births (> 37 gestational weeks and 0 days). Adjustment for gestational age among infants born at term did not changes the results (data not shown).							

No obvious association was found between PFOA, PFNA, PFDA, and PFUnA and birth weight (Table 3) regardless of whether exposures were modeled as continuous or categorical variables. For individual compounds the direction of the estimates was not consistent in each quartile, and no dose–response relationships were evident.

Restricted cubic splines of the associations between PFAAs and birth weight are presented in Figure S3. In general these did not identify any clear thresholds nor did they indicate monotonic dose–response patterns.

The associations between PFAA exposure and birth weight did not differ substantially between boys and girls for most compounds (Table 4). For PFNA and PFDA, estimated mean birth weights were lower for girls than for boys for quartiles above the first quartile, but in girls there was no monotonic dose–response pattern, and some of the apparent sex differences were attributable to higher estimated mean birth weight in boys with exposures above the lowest quartile.

**Table 4:** Sex-stratified associations between levels of perfluoroalkyl acids and birth weight in 764 boys and 743 girls from the Aarhus Birth Cohort, 2008–2013.

PFAA/exposure scale	Birth weight in boys		Birth weight in girls		Sex-exposure interaction^*b*^
	Crude	Adjusted^*a*^ (95% CI)	Crude	Adjusted^*a*^ (95% CI)	Adjusted^*a*^ (95% CI)
PFHxS
Q1	Reference	Reference	Reference	Reference	Reference
Q2	–14	–21 (–124, 82)	5	6 (–93, 106)	27 (–115, 170)
Q3	–31	–19 (–127, 89)	–19	–21 (–123, 80)	–3 (–151, 145)
Q4	–13	–16 (–125, 93)	–56	–34 (–141, 73)	–17 (–170, 135)
Per IQR (0.3 ng/mL)	–14	–14 (–51, 22)	–5	–1 (–25, 22)	13 (–30, 56)
Per 0.1 ng/mL	–5	–5 (–19, 8)	–2	0 (–9, 8)	5 (–11, 21)
PFHpS
Q1	Reference	Reference	Reference	Reference	Reference
Q2	–82	–69 (–178, 40)	–36	–39 (–138, 60)	30 (–117, 177)
Q3	0	0 (–102, 101)	–48	–60 (–153, 37)	–58 (–197, 82)
Q4	–15	–16 (–123, 92)	–76	–78 (–176, 20)	–63 (–208, 83)
Per IQR (0.1 ng/mL)	5	4 (–35, 43)	–27	–30 (–72, 12)	–34 (–91, 23)
Per 0.1 ng/mL	5	4 (–32, 39)	–25	–27 (–65, 11)	–31 (–83, 21)
PFOS
Q1	Reference	Reference	Reference	Reference	Reference
Q2	–132	–129 (–239, –19)	–37	–44 (–140, 52)	85 (–61, 231)
Q3	16	9 (–93, 110)	–56	–55 (–148, 38)	–63 (–201, 75)
Q4	–40	–37 (–141, 67)	–73	–71 (–174, 31)	–34 (–180, 112)
Per IQR (4.8 ng/mL)	25	26 (–13, 65)	–32	–32 (–71, 7)	–58 (–114, –3)
Per 0.1 ng/mL	1	1 (0, 1)	–1	–1 (–1, 0)	–1 (–2, –1)
PFOA
Q1	Reference	Reference	Reference	Reference	Reference
Q2	–35	–17 (–126, 93)	–11	6 (–95, 107)	23 (–126, 172)
Q3	36	54 (–54, 163)	–11	–2 (–100, 95)	–56 (–202, 89)
Q4	11	21 (–84, 126)	11	22 (–79, 124)	1 (–144, 147)
Per IQR (1.1 ng/mL)	27	31 (4, 59)	1	4 (–34, 42)	–27 (–74, 20)
Per 0.1 ng/mL	2	3 (0, 5)	0	0 (–3, 4)	–2 (–7, 2)
PFNA
Q1	Reference	Reference	Reference	Reference	Reference
Q2	63	62 (–48, 172)	–89	–97 (–193, –2)	–160 (–305, –14)
Q3	20	19 (–94, 133)	–131	–123 (–218, –29)	–143 (–291, 5)
Q4	50	46 (–64, 155)	–39	–35 (–133, 63)	–81 (–228, 66)
Per IQR (0.4 ng/mL)	28	27 (–5, 59)	–5	–4 (–38, 31)	–31 (–78, 16)
Per 0.1 ng/mL	8	7 (–2, 16)	–1	–1 (–11, 8)	–9 (–22, 5)
PFDA
Q1	Reference	Reference	Reference	Reference	Reference
Q2	11	13 (–98, 124)	–65	–70 (–167, 26)	–84 (–230, 63)
Q3	30	38 (–73, 148)	–136	–127 (–228, –26)	–165 (–314, –15)
Q4	74	84 (–25, 192)	–73	–58 (–156, 39)	–142 (–287, 4)
Per IQR (0.2 ng/mL)	18	21 (–7, 49)	–6	0 (–36, 37)	–20 (–166, 25)
Per 0.1 ng/mL	10	12 (–4, 27)	–3	0 (–20, 21)	–11 (–37, 14)
PFUnA
Q1	Reference	Reference	Reference	Reference	Reference
Q2	–17	–12 (–124, 99)	–14	0 (–97, 97)	13 (–135, 161)
Q3	7	25 (–86, 136)	2	24 (–79, 127)	–1 (–152, 150)
Q4	–19	13 (–101, 127)	–41	–11 (–102, 81)	–24 (–169, 122)
Per IQR (0.2 ng/mL)	–8	1 (–33, 35)	–13	–5 (–31, 22)	–6 (–49, 37)
Per 0.1 ng/mL	–3	1 (–14, 15)	–5	–2 (–13, 9)	–2 (–20, 15)
Abbreviations: IQR, interquartile range; Q, quartile. For PFAA abbreviations, see Table 1. ^***a***^Adjusted for maternal age, prepregnancy BMI, and educational level. ^***b***^Differences in birth weight between sexes are based on girls compared with boys—negative estimates refer to lower birth weight estimates for girls compared with boys.					

Estimates for the association between PFAA exposures and birth length and head circumference at birth were all close to zero (see Table S3). There was no obvious association between PFAA exposure and gestational age at birth or preterm birth (see Table S4). The levels of PFAAs were similar in samples taken before and after 13 gestational weeks (data not shown), and the exclusion of 67 participants with a blood sample drawn after 13 completed gestational weeks did not change the results (data not shown).

## Discussion

Overall, we found weak and inconsistent associations between PFAA exposures and birth weight, birth length, and head circumference at birth. Independent of offspring sex, estimated mean birth weights were lower in quartiles above the lowest quartile of three perfluoroalkane sulfonic acids, without clear monotonic dose–response patterns, whereas PFNA and PFDA tended to be associated with lower birth weight in girls only. However, the sex-specific estimates were less precise than the estimates from the complete population.

Quite a few studies have investigated the association between PFAAs and infant size at birth. PFAAs were measured in plasma or serum from maternal blood before conception, during pregnancy, or at birth, or in umbilical cord blood; and exposures were modeled as different continuous or categorical variables, which have made comparison of the results cumbersome. Also, the studies controlled for different factors (Bach et al. 2015). Nine studies investigated the association between PFOA and birth weight (Apelberg et al. 2007; Chen et al. 2012; Darrow et al. 2013; Fei et al. 2007; Hamm et al. 2010; Kishi et al. 2015; Maisonet et al. 2012; Robledo et al. 2015; Washino et al. 2009), and all found lower estimated mean birth weights with increasing exposure levels even though the magnitude and precision of the estimates varied substantially. Seven of 10 studies found tendencies towards lower birth weight with higher PFOS levels (Apelberg et al. 2007; Chen et al. 2012; Darrow et al. 2013; Fei et al. 2007; Hamm et al. 2010; Inoue et al. 2004; Kishi et al. 2015; Maisonet et al. 2012; Robledo et al. 2015; Washino et al. 2009). Compared with some previous studies, we report lower serum levels of PFOS (Darrow et al. 2013; Fei et al. 2007; Lee et al. 2013; Maisonet et al. 2012; Monroy et al. 2008) and PFOA (Darrow et al. 2013; Fei et al. 2007; Maisonet et al. 2012; Wu et al. 2012). For instance, the mean level of PFOA was 5.6 ng/mL in a previous Danish study (1996–2002) by Fei et al. (2007) compared with 2.2 ng/mL in the present study. In the study by Fei et al. (2007), the mean level of PFOS was 35.3 ng/mL compared with 8.9 ng/mL in the present study. This may be attributable to a decreasing exposure trend over time. If a threshold value of exposure exists, this may partly explain the lack of an association between PFOA and birth weight, birth length, and head circumference at birth in the present study. It is possible that regulatory measures might have decreased exposure sources sufficiently, perhaps to an extent that PFOA does not pose a potential threat to perinatal health. Thus, the results of the present study are largely reassuring. However, in accordance with other studies, the present results suggest an association between PFOS and lower birth weight. Other compounds, such as PFHxS and PFHpS, were also associated with lower birth weight. If an association between PFAAs and birth weight is mediated by changes in the sex hormone homeostasis, this may explain the somewhat different associations that we found according to offspring sex for PFNA and PFDA, but these differences may also be attributable to statistical imprecision. Compounds other than PFOS and PFOA have been studied to a lesser extent, are currently not regulated, and thus need further investigation.

We studied women who were nulliparous at inclusion, gave birth to a live-born child, provided blood samples, and completed a questionnaire. This may influence the validity and generalizability of our results. Selection bias due to nonresponse may not be very likely since participants were unaware of individual exposure levels (at any time) as well as the outcomes at the time of inclusion. However, it cannot be ruled out that the selection of study participants may have been associated with both exposure levels and the outcomes.

A few studies investigated the association between PFAAs and fecundability (Buck Louis et al. 2013; Fei et al. 2009; Jørgensen et al. 2014; Vélez et al. 2015; Vestergaard et al. 2012; Whitworth et al. 2012a), miscarriage (Darrow et al. 2014; Jensen et al. 2015; Savitz et al. 2012a; Stein et al. 2009), and stillbirth (Savitz et al. 2012a, 2012b) and found inconsistent results. Survival until birth may be less likely to occur among smaller fetuses, and if PFAA exposure is also associated with a decreased chance of live birth, restriction of the study population to live births only may have introduced selection bias which potentially attenuated a possible association between PFAAs and lower birth weight (Liew et al. 2015). However, the magnitude of a possible bias is largely unknown, and the bias relies on the strong assumption of very strong associations between PFAA exposure and a decreased chance of live birth. Adjustment for common causes of low birth weight and fetal survival, such as maternal age, BMI, and educational level, would have reduced a possible live birth bias (Liew et al. 2015).

Accurate exposure assessment and the use of multiple exposure scales (continuous, categorical, splines) were strengths of our study. We chose to use the same continuous exposure scales for all compounds even though an increase of 0.1 ng/mL was a large increase for compounds with low average concentrations and a small increase for compounds with higher average concentrations such as PFOS and PFOA. To ease comparability for the different PFAAs within our study, we also used continuous exposures divided by the interquartile range. We demanded strict laboratory procedures from sampling to the final analysis. Limited time was allowed from blood sampling to processing and freezing, state-of-the-art laboratory equipment was used, and the high performance chromatography–tandem mass spectrometry setup was controlled using internal and external validation and controls. Objectively measured exposures such as PFAA levels are unlikely to be prone to differential measurement error. Because levels of PFAAs decrease throughout pregnancy (Glynn et al. 2012), we only included women who gave a blood sample before 20 completed gestational weeks.

Trained health care professionals systematically assessed the outcomes as part of routine data collection concerning all births at the hospital. Measurement error concerning birth weight, particularly differential measurement error, is unlikely to be of importance. However, birth length and head circumference at birth may be more prone to measurement error, and this might partly explain our close-to-null results for these outcomes.

We were able to control for the potential confounders we considered to be most important (see Figures S1 and S2), including maternal prepregnancy BMI, age, educational level, and parity (by restriction to first-time mothers). Maternal prepregnancy BMI and education were self-reported and may thus be recorded with some error. It is debatable whether the slightly stronger association for PFHxS, PFHpS, and PFOS after adjustment for gestational age, restriction to term births, or modeling of *z*-scores may be attributable to collider stratification bias (if gestational age is a mediator of the association between PFAAs and birth weight, and any factors we did not account for affected both gestational age and birth weight, conditioning on gestational age may have induced a spurious association between PFAAs and birth weight).

Physiological phenomena in pregnancy, including changes in the maternal glomerular filtration rate (GFR) and plasma volume expansion, may be potentially important confounders if exposures are assessed in late pregnancy or at birth (Loccisano et al. 2013; Morken et al. 2014). These phenomena are of much larger magnitude in late than in early pregnancy, and the fact that we measured PFAA levels early is likely to have reduced potential confounding by these factors, which we did not collect information on. In previous studies measuring PFAAs in late pregnancy, or at birth, confounding by changes in GFR and plasma volume expansion may potentially explain the associations demonstrated between higher levels of PFAAs and lower birth weight. However, in a recent review of the literature, we found no systematic differences in the magnitude and direction of the association between PFAAs and the estimated mean birth weight differences according to the timing of exposure assessment (Bach et al. 2015). Because the causal window of exposure is largely unknown, the optimal timing of exposure assessment is unsettled. We consider early measurements to be preferable in order to limit the impact of physiological changes during pregnancy that may influence the measured exposures.

Levels of PFAA compounds are correlated (see Table S2), and different PFAAs are likely derived from similar exposure sources. Biologically, it is possible that different PFAAs share health effects.

## Conclusions

We found no strong associations between PFAA exposures and birth weight, birth length, or head circumference at birth. In particular, we failed to replicate the association between PFOA and lower birth weight previously shown primarily in populations with higher exposures that were not restricted to nulliparous women. The estimated mean birth weights were lower in quartiles above the reference for PFOS, PFHxS, and PFHpS for all infants and for PFNA and PFDA in girls only. Most of the compounds we investigated have not been studied much, and more studies are warranted.

## Supplemental Material

(721 KB) PDF

## References

[r1] Abbott BD 2009 Review of the expression of peroxisome proliferator-activated receptors alpha (PPARα), beta (PPARβ), and gamma (PPARγ) in rodent and human development. Reprod Toxicol 27 246 257, doi:10.1016/j.reprotox.2008.10.001 18996469

[r2] Alexander GR Himes JH Kaufman RB Mor J Kogan M 1996 A United States national reference for fetal growth. Obstet Gynecol 87 163 168, doi:10.1016/0029-7844(95)00386-X 8559516

[r3] Andersen CS Fei C Gamborg M Nohr EA Sørensen TIA Olsen J 2010 Prenatal exposures to perfluorinated chemicals and anthropometric measures in infancy. Am J Epidemiol 172 1230 1237, doi:10.1093/aje/kwq289 20940176

[r4] Apelberg BJ Witter FR Herbstman JB Calafat AM Halden RU Needham LL 2007 Cord serum concentrations of perfluorooctane sulfonate (PFOS) and perfluorooctanoate (PFOA) in relation to weight and size at birth. Environ Health Perspect 115 1670 1676, doi:10.1289/ehp.10334 18008002 PMC2072847

[r5] Arbuckle TE Kubwabo C Walker M Davis K Lalonde K Kosarac I 2013 Umbilical cord blood levels of perfluoroalkyl acids and polybrominated flame retardants. Int J Hyg Environ Health 216 184 194, doi:10.1016/j.ijheh.2012.03.004 22494936

[r6] Bach CC Bech BH Brix N Nohr EA Bonde JPE Henriksen TB 2015 Perfluoroalkyl and polyfluoroalkyl substances and human fetal growth: a systematic review. Crit Rev Toxicol 45 53 67, doi:10.3109/10408444.2014.952400 25372700

[r7] Benninghoff AD, Bisson WH, Koch DC, Ehresman DJ, Kolluri SK, Williams DE (2011). Estrogen-like activity of perfluoroalkyl acids *in vivo* and interaction with human and rainbow trout estrogen receptors *in vitro*.. Toxicol Sci.

[r8] Buck Louis GM Sundaram R Schisterman EF Sweeney AM Lynch CD Gore-Langton RE 2013 Persistent environmental pollutants and couple fecundity: the LIFE Study. Environ Health Perspect 121 231 236, doi:10.1289/ehp.1205301 23151773 PMC3569685

[r9] Butenhoff JL Olsen GW Pfahles-Hutchens A 2006 The applicability of biomonitoring data for perfluorooctanesulfonate to the environmental public health continuum. Environ Health Perspect 114 1776 1782, doi:10.1289/ehp.9060 17107867 PMC1665413

[r10] Chen MH Ha EH Wen TW Su YN Lien GW Chen CY 2012 Perfluorinated compounds in umbilical cord blood and adverse birth outcomes. PLoS One 7 e42474, doi:10.1371/journal.pone.0042474 22879996 PMC3411780

[r11] Darrow LA Howards PP Winquist A Steenland K 2014 PFOA and PFOS serum levels and miscarriage risk. Epidemiology 25 505 512, doi:10.1097/EDE.0000000000000103 24807698

[r12] Darrow LA Stein CR Steenland K 2013 Serum perfluorooctanoic acid and perfluorooctane sulfonate concentrations in relation to birth outcomes in the Mid-Ohio Valley, 2005–2010. Environ Health Perspect 121 1207 1213, doi:10.1289/ehp.1206372 23838280 PMC3801459

[r13] Du G Hu J Huang H Qin Y Han X Wu D 2013 Perfluorooctane sulfonate (PFOS) affects hormone receptor activity, steroidogenesis, and expression of endocrine-related genes in vitro and in vivo. Environ Toxicol Chem 32 353 360, doi:10.1002/etc.2034 23074026

[r14] Environment Canada (2010). Environmental Performance Agreement (“Agreement”) Respecting Perfluorinated Carboxylic Acids (Pfcas) and their Precursors in Perfluorochemical Products Sold in Canada.. http://www.ec.gc.ca/epe-epa/AE06B51E-7BB7-4C92-A4B5-6027E9195712/PFCA_EPA-EN.pdf.

[r15] European Parliament (2006). Directive 2006/122/EC of the European Parliament and of the Council of 12 December 2006 amending for the 30th time Council Directive 76/769/EEC on the approximation of the laws, regulations and administrative provisions of the Member States relating to restrictions on the marketing and use of certain dangerous substances and preparations (perfluorooctane sulfonates).. Official Journal of the European Union L372/32-L372/34.

[r16] Fei C McLaughlin JK Lipworth L Olsen J 2009 Maternal levels of perfluorinated chemicals and subfecundity. Hum Reprod 24 1200 1205, doi:10.1093/humrep/den490 19176540

[r17] Fei C McLaughlin JK Tarone RE Olsen J 2007 Perfluorinated chemicals and fetal growth: a study within the Danish National Birth Cohort. Environ Health Perspect 115 1677 1682, doi:10.1289/ehp.10506 18008003 PMC2072850

[r18] Glynn A Berger U Bignert A Ullah S Aune M Lignell S 2012 Perfluorinated alkyl acids in blood serum from primiparous women in Sweden: serial sampling during pregnancy and nursing, and temporal trends 1996-2010. Environ Sci Technol 46 16 9071 9079, doi:10.1021/es301168c 22770559

[r19] Hamm MP Cherry NM Chan E Martin JW Burstyn I 2010 Maternal exposure to perfluorinated acids and fetal growth. J Expo Sci Environ Epidemiol 20 589 597, doi:10.1038/jes.2009.57 19865074

[r20] Henry ND, Fair PA (2011). Comparison of *in vitro* cytotoxicity, estrogenicity and anti-estrogenicity of triclosan, perfluorooctane sulfonate and perfluorooctanoic acid.. J Appl Toxicol.

[r21] Inoue K Okada F Ito R Kato S Sasaki S Nakajima S 2004 Perfluorooctane sulfonate (PFOS) and related perfluorinated compounds in human maternal and cord blood samples: assessment of PFOS exposure in a susceptible population during pregnancy. Environ Health Perspect 112 1204 1207, doi:10.1289/ehp.6864 15289168 PMC1247483

[r22] Jensen TK Andersen LB Kyhl HB Nielsen F Christesen HT Grandjean P 2015 Association between perfluorinated compound exposure and miscarriage in Danish pregnant women. PloS One 10 e0123496, doi:10.1371/journal.pone.0123496 25848775 PMC4388566

[r23] Jørgensen KT Specht IO Lenters V Bach CC Rylander L Jönsson BA 2014 Perfluoroalkyl substances and time to pregnancy in couples from Greenland, Poland and Ukraine. Environ Health 13 116, doi:10.1186/1476-069X-13-116 25533644 PMC4391306

[r24] Kantiani L Llorca M Sanchís J Farré M Barceló D 2010 Emerging food contaminants: a review. Anal Bioanal Chem 398 2413 2427, doi:10.1007/s00216-010-3944-9 20680618

[r25] Kim S Choi K Ji K Seo J Kho Y Park J 2011 Trans-placental transfer of thirteen perfluorinated compounds and relations with fetal thyroid hormones. Environ Sci Technol 45 7465 7472, doi:10.1021/es202408a 21805959

[r26] Kishi R Nakajima T Goudarzi H Kobayashi S Sasaki S Okada E 2015 The association of prenatal exposure to perfluorinated chemicals with maternal essential and long-chain polyunsaturated fatty acids during pregnancy and the birth weight of their offspring: the Hokkaido Study. Environ Health Perspect 123 1038 1045, doi:10.1289/ehp.1408834 25840032 PMC4590753

[r27] Kjeldsen LS Bonefeld-Jørgensen EC 2013 Perfluorinated compounds affect the function of sex hormone receptors. Environ Sci Pollut Res Int 20 8031 8044, doi:10.1007/s11356-013-1753-3 23764977

[r28] Koustas E Lam J Sutton P Johnson PI Atchley DS Sen S 2014 The Navigation Guide—evidence-based medicine meets environmental health: systematic review of nonhuman evidence for PFOA effects on fetal growth. Environ Health Perspect 122 1015 1027, doi:10.1289/ehp.1307177 24968374 PMC4181920

[r29] Larsen PS Kamper-Jørgensen M Adamson A Barros H Bonde JP Brescianini S 2013 Pregnancy and birth cohort resources in Europe: a large opportunity for aetiological child health research. Paediatr Perinat Epidemiol 27 393 414, doi:10.1111/ppe.12060 23772942

[r30] Lau C Thibodeaux JR Hanson RG Rogers JM Grey BE Stanton ME 2003 Exposure to perfluorooctane sulfonate during pregnancy in rat and mouse. II postnatal evaluation Toxicol Sci 74 382 392, doi:10.1093/toxsci/kfg122 12773772

[r31] Lee YJ Kim MK Bae J Yang JH 2013 Concentrations of perfluoroalkyl compounds in maternal and umbilical cord sera and birth outcomes in Korea. Chemosphere 90 1603 1609, doi:10.1016/j.chemosphere.2012.08.035 22990023

[r32] Liew Z Olsen J Cui X Ritz B Arah OA 2015 Bias from conditioning on live birth in pregnancy cohorts: an illustration based on neurodevelopment in children after prenatal exposure to organic pollutants. Int J Epidemiol 44 345 354, doi:10.1093/ije/dyu249 25604449 PMC4339763

[r33] Liew Z Ritz B Bonefeld-Jørgensen EC Henriksen TB Nohr EA Bech BH 2014 Prenatal exposure to perfluoroalkyl substances and the risk of congenital cerebral palsy in children. Am J Epidemiol 180 574 581, doi:10.1093/aje/kwu179 25139206 PMC12118604

[r34] Loccisano AE Longnecker MP Campbell JL Jr Andersen ME Clewell HJ III 2013 Development of PBPK models for PFOA and PFOS for human pregnancy and lactation life stages. J Toxicol Environ Health A 76 1 25 57, doi:10.1080/15287394.2012.722523 23151209 PMC3502013

[r35] Long M Ghisari M Bonefeld-Jørgensen EC 2013 Effects of perfluoroalkyl acids on the function of the thyroid hormone and the aryl hydrocarbon receptor. Environ Sci Pollut Res Int 20 8045 8056, doi:10.1007/s11356-013-1628-7 23539207

[r36] Luebker DJ, York RG, Hansen KJ, Moore JA, Butenhoff JL (2005). Neonatal mortality from in utero exposure to perfluorooctane sulfonate (PFOS) in Sprague–Dawley rats: dose–response, and biochemical and pharmacokinetic parameters.. Toxicology.

[r37] Maisonet M Terrell ML McGeehin MA Christensen KY Holmes A Calafat AM 2012 Maternal concentrations of polyfluoroalkyl compounds during pregnancy and fetal and postnatal growth in British girls. Environ Health Perspect 120 1432 1437, doi:10.1289/ehp.1003096 22935244 PMC3491920

[r38] Marsál K, Persson PH, Larsen T, Lilja H, Selbing A, Sultan B (1996). Intrauterine growth curves based on ultrasonically estimated foetal weights.. Acta Paediatr.

[r39] Midasch O Drexler H Hart N Beckmann MW Angerer J 2007 Transplacental exposure of neonates to perfluorooctanesulfonate and perfluorooctanoate: a pilot study. Int Arch Occup Environ Health 80 643 648, doi:10.1007/s00420-006-0165-9 17219182

[r40] Monroy R Morrison K Teo K Atkinson S Kubwabo C Stewart B 2008 Serum levels of perfluoroalkyl compounds in human maternal and umbilical cord blood samples. Environ Res 108 56 62, doi:10.1016/j.envres.2008.06.001 18649879

[r41] Morken NH Travlos GS Wilson RE Eggesbø M Longnecker MP 2014 Maternal glomerular filtration rate in pregnancy and fetal size. PLoS One 9 7 e101897, doi:10.1371/journal.pone.0101897 25003331 PMC4087025

[r42] Mortensen LM Bech BH Nohr EA Kruhøffer M Kjærgaard S Uldbjerg N 2013 Data resource profile: the Aarhus Birth Cohort Biobank (ABC Biobank). Int J Epidemiol 42 1697 1701, doi:10.1093/ije/dyt199 24415608

[r43] Robledo CA Yeung E Mendola P Sundaram R Maisog J Sweeney AM 2015 Preconception maternal and paternal exposure to persistent organic pollutants and birth size: the LIFE Study. Environ Health Perspect 123 88 94, doi:10.1289/ehp.1308016 25095280 PMC4286275

[r44] Savitz DA Stein CR Bartell SM Elston B Gong J Shin HM 2012a Perfluorooctanoic acid exposure and pregnancy outcome in a highly exposed community. Epidemiology 23 386 392, doi:10.1097/EDE.0b013e31824cb93b 22370857 PMC3321117

[r45] Savitz DA Stein CR Elston B Wellenius GA Bartell SM Shin HM 2012b Relationship of perfluorooctanoic acid exposure to pregnancy outcome based on birth records in the mid-Ohio Valley. Environ Health Perspect 120 1201 1207, doi:10.1289/ehp.1104752 22450153 PMC3440089

[r46] Stein CR Savitz DA Dougan M 2009 Serum levels of perfluorooctanoic acid and perfluorooctane sulfonate and pregnancy outcome. Am J Epidemiol 170 837 846, doi:10.1093/aje/kwp212 19692329

[r47] Takahashi M, Ishida S, Hirata-Koizumi M, Ono A, Hirose A (2014). Repeated dose and reproductive/developmental toxicity of perfluoroundecanoic acid in rats.. J Toxicol Sci.

[r48] Thibodeaux JR Hanson RG Rogers JM Grey BE Barbee BD Richards JH 2003 Exposure to perfluorooctane sulfonate during pregnancy in rat and mouse. I: maternal and prenatal evaluations. Toxicol Sci 74 369 381, doi:10.1093/toxsci/kfg121 12773773

[r49] UNEP (United Nations Environment Programme) (2009). Governments Unite to Step-up Reduction on Global DDT Reliance and Add Nine New Chemicals under International Treaty.. http://chm.pops.int/Convention/Pressrelease/COP4Geneva8May2009/tabid/542/language/en-US/Default.aspx.

[r50] U.S. EPA (U.S. Environmental Protection Agency) (2000). EPA and 3M Announce Phase Out of PFOS.. http://yosemite.epa.gov/opa/admpress.nsf/0/33aa946e6cb11f35852568e1005246b4.

[r51] U.S. EPA (2006). 2010/2015 PFOA Stewardship Program.. http://www.epa.gov/assessing-and-managing-chemicals-under-tsca/20102015-pfoa-stewardship-program.

[r52] Vélez MP Arbuckle TE Fraser WD 2015 Maternal exposure to perfluorinated chemicals and reduced fecundity: the MIREC Study. Hum Reprod 30 701 709, doi:10.1093/humrep/deu350 25567616 PMC4325673

[r53] Vestergaard S Nielsen F Andersson AM Hjøllund NH Grandjean P Andersen HR 2012 Association between perfluorinated compounds and time to pregnancy in a prospective cohort of Danish couples attempting to conceive. Hum Reprod 27 873 880, doi:10.1093/humrep/der450 22246448

[r54] Wang Y Starling AP Haug LS Eggesbo M Becher G Thomsen C 2013 Association between perfluoroalkyl substances and thyroid stimulating hormone among pregnant women: a cross-sectional study. Environ Health 12 76, doi:10.1186/1476-069X-12-76 24010716 PMC3847507

[r55] Washino N Saijo Y Sasaki S Kato S Ban S Konishi K 2009 Correlations between prenatal exposure to perfluorinated chemicals and reduced fetal growth. Environ Health Perspect 117 660 667, doi:10.1289/ehp.11681 19440508 PMC2679613

[r56] Whitworth KW Haug LS Baird DD Becher G Hoppin JA Skjaerven R 2012a Perfluorinated compounds and subfecundity in pregnant women. Epidemiology 23 257 263, doi:10.1097/EDE.0b013e31823b5031 22081060 PMC3276687

[r57] Whitworth KW Haug LS Baird DD Becher G Hoppin JA Skjaerven R 2012b Perfluorinated compounds in relation to birth weight in the Norwegian Mother and Child Cohort Study. Am J Epidemiol 175 1209 1216, doi:10.1093/aje/kwr459 22517810 PMC3372312

[r58] Wilcox AJ Weinberg CR Basso O 2011 On the pitfalls of adjusting for gestational age at birth. Am J Epidemiol 174 1062 1068, doi:10.1093/aje/kwr230 21946386 PMC3243938

[r59] Wu K Xu X Peng L Liu J Guo Y Huo X 2012 Association between maternal exposure to perfluorooctanoic acid (PFOA) from electronic waste recycling and neonatal health outcomes. Environ Int 48 1 8, doi:10.1016/j.envint.2012.06.018 22820015

