# Supplementary material for: Perfluoroalkyl Acids in Maternal Serum and Indices of Fetal Growth: The Aarhus Birth Cohort
Source: Environ Health Perspect. 2015 Oct 23;124(6):848–54. doi: 10.1289/ehp.1510046 (PMC4892925; doi:10.1289/ehp.1510046)
Supplement: (721 KB) PDF [file ehp.1510046.s001.acco.pdf]

Environ Health Perspect
DOI: 10.1289/ehp.1510046

Note to readers with disabilities: *EHP* strives to ensure that all journal content is accessible to all readers. However, some figures and Supplemental Material published in *EHP* articles may not conform to 508 standards due to the complexity of the information being presented. If you need assistance accessing journal content, please contact ehp508@niehs.nih.gov. Our staff will work with you to assess and meet your accessibility needs within 3 working days.

Supplemental Material

Perfluoroalkyl Acids in Maternal Serum and Indices of Fetal Growth: The Aarhus Birth Cohort

Cathrine Carlsen Bach, Bodil Hammer Bech, Ellen Aagaard Nohr, Jørn Olsen, Niels Bjerregård Matthiesen, Eva Cecilie Bonefeld-Jørgensen, Rossana Bossi, and Tine Brink Henriksen

Table of Contents

Figure S1. Directed acyclic graph illustrating the association between perfluoroalkyl acid (PFAA) exposure and birth weight. The figure also applies to the outcomes birth length and head circumference at birth. In the main analyses we conditioned on the variables marked with white circles. According to this graph it was not necessary to condition on the variables marked with grey circles. Figure made by use of daggity.net (Textor et al. 2011).

Figure S2. Directed acyclic graph illustrating the association between perfluoroalkyl acid (PFAA) exposure and preterm birth. The illustration also applies to the continuous gestational age outcome. In the main analyses we conditioned on the variables marked with white circles. According to this graph it was not necessary to condition on the variables marked with grey circles. Figure made by use of daggity.net (Textor et al. 2011).

Figure S3. Restricted cubic splines with pre-specified knots according to quartile boundaries of PFAAs. Illustrates the association between individual PFAAs and birth weight, adjusted for maternal pre-pregnancy body mass index, educational level, and age. The shading indicates 95 % confidence intervals. See PFAA abbreviations in Table 1 in the main document.

Table S1. Perfluoroalkyl acid abbreviations, limits of quantification (LOQ), and levels above the

LOQ for compounds not included in the study of 1507 women from the Aarhus Birth Cohort, 2008-2013.

Table S2. Pearson's correlations for perfluoroalkyl acids in 1507 women from the Aarhus Birth Cohort, 2008-2013.

Table S3. Levels of perfluoroalkyl acids, birth length (n= 1499) and head circumference at birth (n=1494) in the Aarhus Birth Cohort, 2008-2013.

Table S4. Levels of perfluoroalkyl acids and gestational length at birth or preterm birth in 1507 children from the Aarhus Birth Cohort, 2008-2013.

References

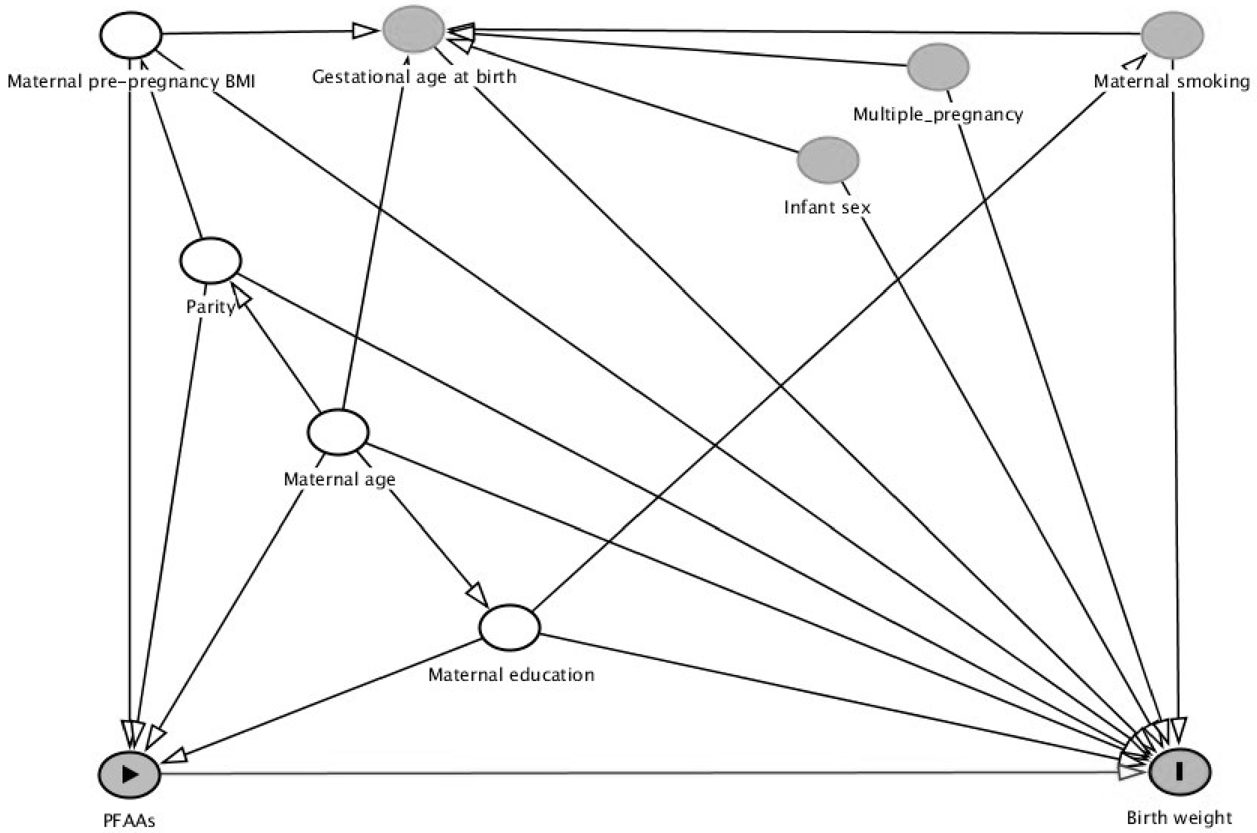

Figure S1. Directed acyclic graph illustrating the association between perfluoroalkyl acid (PFAA) exposure and birth weight. The figure also applies to the outcomes birth length and head circumference at birth. In the main analyses we conditioned on the variables marked with white circles. According to this graph it was not necessary to condition on the variables marked with grey circles. Figure made by use of daggity.net (Textor et al. 2011).

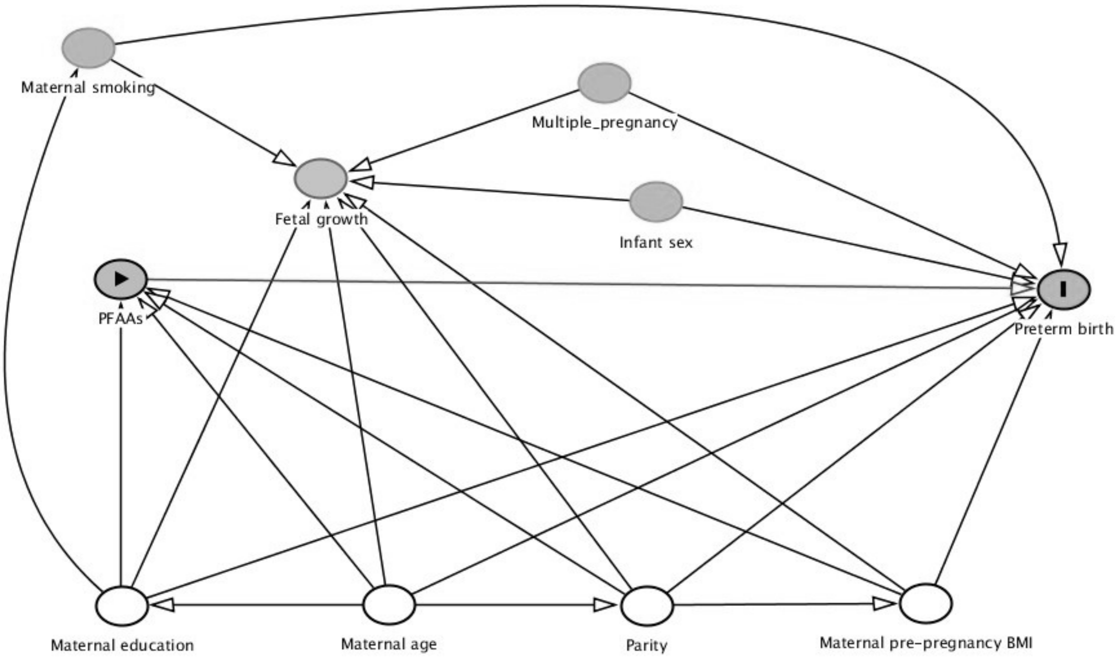

Figure S2. Directed acyclic graph illustrating the association between perfluoroalkyl acid (PFAA) exposure and preterm birth. The illustration also applies to the continuous gestational age outcome. In the main analyses we conditioned on the variables marked with white circles. According to this graph it was not necessary to condition on the variables marked with grey circles. Figure made by use of daggity.net (Textor et al. 2011).

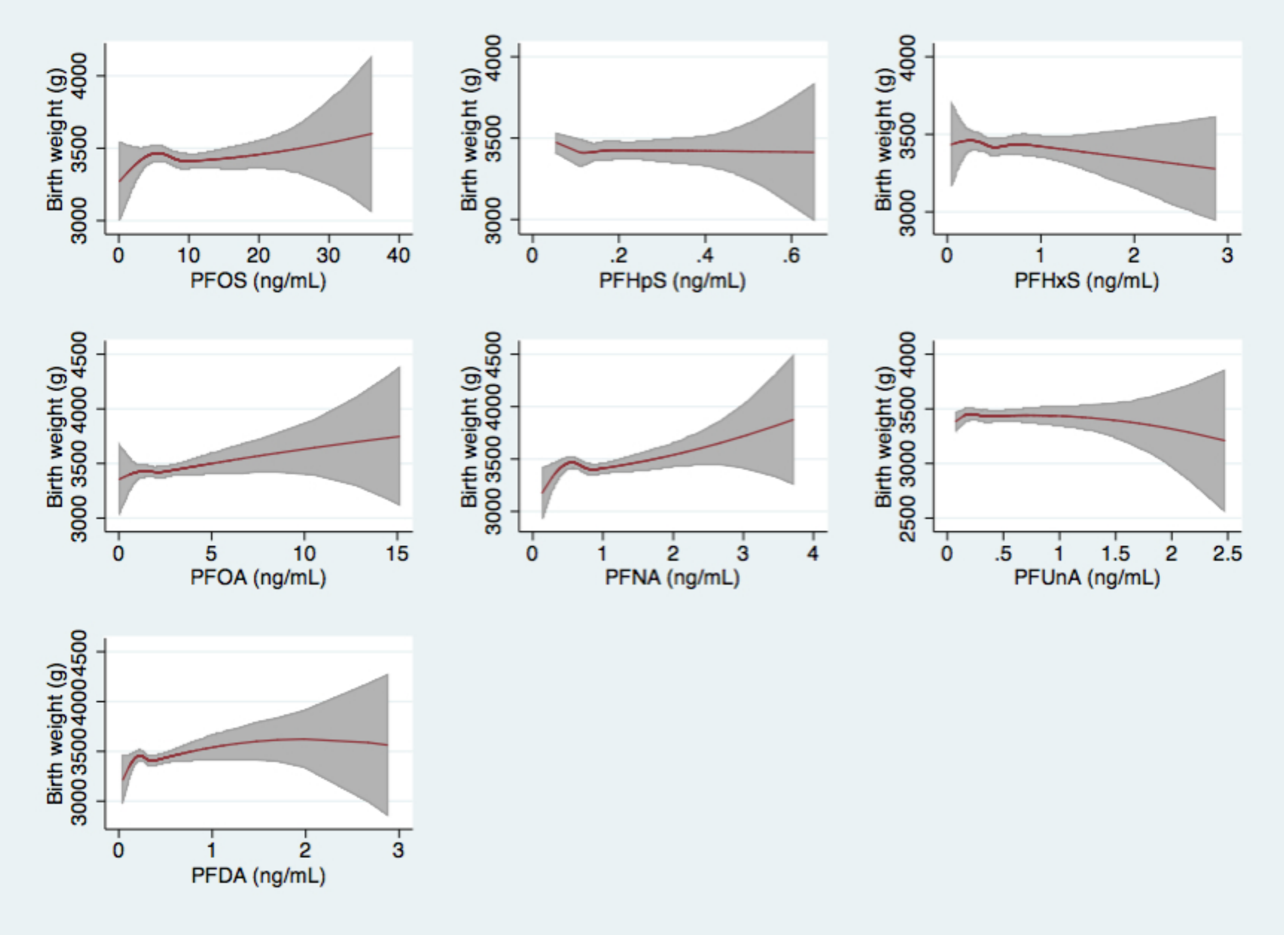

Figure S3. Restricted cubic splines with pre-specified knots according to quartile boundaries of PFAAs. Illustrates the association between individual PFAAs and birth weight, adjusted for maternal pre-pregnancy body mass index, educational level, and age. The shading indicates 95 % confidence intervals. See PFAA abbreviations in Table 1 in the main document.

Table S1. Perfluoroalkyl acid abbreviations, limits of quantification (LOQ), and levels above the LOQ for compounds not included in the study of 1507 women from the Aarhus Birth Cohort, 2008-2013.

**Full name**	**Abbreviation**	**LOQ** ng/mL	**% above LOQ**
Perfluorobutane sulfonate	PFBS	0.07	1
Perfluorodecane sulfonate	PFDS	0.37	0
Perfluorooctane sulfonamide	PFOSA	1.19	0
Perfluoropentanoic acid	PFPeA	0.19	6
Perfluorohexanoic acid	PFHxA	0.03	2
Perfluoroheptanoic acid	PFHpA	0.05	26
Perfluorododecanoic acid	PFDoA	0.41	5
Perfluorotridecanoic acid	PFTrA	0.41	0.3
Perfluorotetradecanoic acid	PFTeA	0.41	0

Table S2. Pearson's correlations for perfluoroalkyl acids in 1507 women from the Aarhus Birth Cohort, 2008-2013.

	PFHxS	PFHpS	PFOS	PFOA	PFNA	PFDA
PFHpS	0.47					
PFOS	0.38	0.80				
PFOA	0.24	0.38	0.40			
PFNA	0.20	0.39	0.46	0.82		
PFDA	0.15	0.3	0.38	0.71	0.85	
PFUnA	0.14	0.31	0.36	0.21	0.40	0.60

See PFAA abbreviations in Table 1 in the main document.

p<0.001 for all correlations.

Table S3. Levels of perfluoroalkyl acids, birth length (n= 1499) and head circumference at birth (n=1494) in the Aarhus Birth Cohort, 2008-2013.

PFAA	Exposure scale	Birth length (cm)			Head circumference (cm)		
		Median (IQR)	Crude	Adjusted	Median (IQR)	Crude	Adjusted
**PFHxS**	Q1	52 (50-53)	Ref.	Ref.	35 (34-36)	Ref.	Ref.
	Q2	52 (50-53)	0.1	0.1 (-0.3, 0.4)	35 (34-36)	-0.1	-0.2 (-0.4, 0.1)
	Q3	52 (50-53)	-0.1	-0.1 (-0.5, 0.3)	35 (34-36)	-0.2	-0.2 (-0.5, 0.0)
	Q4	52 (50-53)	-0.2	-0.2 (-0.5, 0.2)	35 (34-36)	-0.2	-0.2 (-0.5, 0.1)
	Per IQR (0.3 ng/mL)		0.0	0.0 (-0.1, 0.1)		-0.1	0.0 (-0.1, 0.0)
	Per 0.1 ng/mL		0.0	0.0 (0.0, 0.0)		0.0	0.0 (0.0, 0.0)
**PFHpS**	Q1	52 (51-53)	Ref.	Ref.	35 (34-36)	Ref.	Ref.
	Q2	52 (50-53)	-0.3	-0.3 (-0.7, 0.03)	35 (34-36)	-0.1	-0.1 (-0.3, 0.2)
	Q3	52 (50-53)	-0.1	-0.2 (-0.5, 0.2)	35 (34-36)	0.0	0.0 (-0.2, 0.2)
	Q4	52 (50-53)	-0.2	-0.2 (-0.5, 0.2)	35 (34-36)	0.0	0.0 (-0.3, 0.2)
	Per IQR (0.1 ng/mL)		0.0	0.0 (-0.2, 0.1)		0.0	0.0 (-0.1, 0.1)
	Per 0.1 ng/mL		0.0	0.0 (-0.2, 0.1)		0.0	0.0 (-0.1, 0.1)
**PFOS**	Q1	52 (50-53)	Ref.	Ref.	35 (34-36)	Ref.	Ref.
	Q2	52 (50-53)	-0.3	-0.3 (-0.7, 0.0)	35 (34-36)	-0.2	-0.2 (-0.5, 0.0)
	Q3	52 (50-53)	-0.1	-0.1 (-0.4, 0.3)	35 (34-36)	-0.1	-0.1 (-0.4, 0.1)
	Q4	52 (50-53)	-0.1	-0.1 (-0.5, 0.2)	35 (34-36)	-0.1	-0.1 (-0.3, 0.2)
	Per IQR (4.8 ng/mL)		0.0	0.0 (-0.1, 0.2)		0.0	0.0 (-0.1, 0.1)
	Per 0.1 ng/mL		0.0	0.0 (0.0, 0.0)		0.0	0.0 (0.0, 0.0)
**PFOA**	Q1	52 (50-53)	Ref.	Ref.	35 (34-36)	Ref.	Ref.
	Q2	52 (50-53)	-0.1	0.0 (-0.4, 0.4)	35 (34-36)	0.0	0.0 (-0.2, 0.3)
	Q3	52 (50-53)	0.0	0.0 (-0.4, 0.4)	35 (34-36)	0.1	0.1 (-0.2, 0.4)
	Q4	52 (50-53)	0.0	0.1 (-0.3, 0.4)	35 (34-36)	0.1	0.1 (-0.1, 0.4)
	Per IQR (1.1 ng/mL)		0.1	0.1 (-0.1, 0.2)		0.1	0.1 (0.0, 0.2)
	Per 0.1 ng/mL		0.0	0.0 (0.0, 0.0)		0.0	0.0 (0.0, 0.0)
**PFNA**	Q1	52 (50-53)	Ref.	Ref.	35 (34-36)	Ref.	Ref.
	Q2	52 (50-53)	0.1	0.1 (-0.3, 0.4)	35 (34-36)	0.2	0.1 (-0.1, 0.4)
	Q3	52 (50-53)	-0.2	-0.2 (-0.6, 0.2)	35 (34-36)	-0.2	-0.2 (-0.4, 0.1)
	Q4	52 (50-53)	0.1	0.1 (-0.3, 0.4)	35 (34-36)	0.2	0.2 (-0.1, 0.4)
	Per IQR (0.4 ng/mL)		0.0	0.0 (-0.1, 0.1)		0.1	0.1 (0.0, 0.2)
	Per 0.1 ng/mL		0.0	0.0 (0.0, 0.0)		0.0	0.0 (0.0, 0.0)
**PFDA**	Q1	52 (50-53)	Ref.	Ref.	35 (34-36)	Ref.	Ref.
	Q2	52 (50-53)	0.0	0.0 (-0.4, 0.3)	35 (34-36)	0.0	-0.1 (-0.3, 0.2)
	Q3	52 (50-53)	0.2	0.2 (-0.2, 0.6)	35 (34-36)	-0.1	-0.1 (-0.3. 0.2)
	Q4	52 (50-53)	0.1	0.1 (-0.3, 0.5)	35 (34-36)	0.2	0.2 (-0.1, 0.5)
	Per IQR (0.2 ng/mL)		0.0	0.1 (-0.1, 0.2)		0.1	0.1 (0.0, 0.2)
	Per 0.1 ng/mL		0.0	0.0 (0.0, 0.1)		0.1	0.1 (0.1, 0.1)

**PFUnA**	Q1	52 (50-53)	Ref.	Ref.	35 (34-36)	Ref.	Ref.
	Q2	52 (50-53)	0.1	0.2 (-0.2, 0.6)	35 (34-36)	0.0	0.0 (-0.2, 0.3)
	Q3	52 (50-53)	0.2	0.3 (-0.1, 0.6)	35 (34-36)	0.0	0.0 (-0.2, 0.3)
	Q4	52 (50-53)	0.1	0.2 (-0.2, 0.6)	35 (34-36)	-0.1	0.0 (-0.2, 0.3)
	Per IQR (0.2 ng/mL)		0.0	0.1 (-0.1, 0.2)		0.0	0.0 (-0.1, 0.1)
	Per 0.1 ng/mL		0.0	0.0 (0.0, 0.1)		0.0	0.0 (0.0, 0.1)

Abbreviations: Quartile (Q), interquartile range (IQR), reference (Ref). See PFAA abbreviations in Table 1 in the main document. Adjusted for maternal age, pre-pregnancy body mass index, and educational level.

Table S4. Levels of perfluoroalkyl acids and gestational length at birth or preterm birth in 1507 children from the Aarhus Birth Cohort, 2008-2013.

PFAA	Exposure scale	Gestational length (weeks)			OR for Preterm birth	
		Median (IQR)	Crude	Adjusted	Crude	Adjusted
**PFHxS**	Q1	40 (39-41)	Ref.	Ref.	Ref.	Ref.
	Q2	40 (39-41)	0.0	0.0 (-0.3, 0.2)	0.70	0.72 (0.38, 1.37)
	Q3	40 (39-41)	0.0	0.0 (-0.3, 0.2)	0.98	0.97 (0.54, 1.77)
	Q4	40 (39-41)	0.1	0.1 (-0.2, 0.4)	0.70	0.68 (0.35, 1.31)
	Per IQR (0.3 ng/mL)		0.0	0.0 (0.0, 0.1)	0.89	0.87 (0.68, 1.13)
	Per 0.1 ng/mL		0.0	0.0 (0.0, 0.0)	0.96	0.95 (0.87, 1.05)
**PFHpS**	Q1	40 (39-41)	Ref.	Ref.	Ref.	Ref.
	Q2	40 (39-41)	-0.2	-0.2 (-0.5, 0.1)	1.10	1.10 (0.61, 1.97)
	Q3	40 (39-41)	0.1	0.1 (-0.2, 0.3)	0.66	0.67 (0.35, 1.31)
	Q4	40 (39-41)	0.0	0.0 (-0.2, 0.3)	0.76	0.76 (0.40, 1.46)
	Per IQR (0.1 ng/mL)		0.0	0.0 (-0.1, 0.1)	0.93	0.93 (0.65, 1.32)
	Per 0.1 ng/mL		0.0	0.0 (-0.1, 0.1)	0.93	0.93 (0.68 1.29)
**PFOS**	Q1	40 (39-41)	Ref.	Ref.	Ref.	Ref.
	Q2	40 (39-41)	-0.1	-0.1 (-0.4, 0.1)	0.94	0.96 (0.53, 1.74)
	Q3	40 (39-41)	0.1	0.0 (-0.2, 0.3)	0.64	0.65 (0.34, 1.26)
	Q4	40 (39-41)	0.0	0.0 (-0.3, 0.2)	0.82	0.82 (0.44, 1.53)
	Per IQR (4.8 ng/mL)		0.0	0.0 (-0.1, 0.1)	0.84	0.85 (0.60, 1.21)
	Per 0.1 ng/mL		0.0	0.0 (0.0, 0.0)	1.00	1.00 (0.99, 1.00)
**PFOA**	Q1	40 (39-41)	Ref.	Ref.	Ref.	Ref.
	Q2	40 (39-41)	0.0	0.0 (-0.3, 0.2)	0.53	0.51 (0.27, 0.98)
	Q3	40 (39-41)	0.1	0.1 (-0.2, 0.3)	0.66	0.67 (0.36, 1.25)
	Q4	40 (39-41)	0.1	0.1 (-0.2, 0.4)	0.77	0.77 (0.42, 1.39)
	Per IQR (1.1 ng/mL)		0.1	0.1 (0.0, 0.2)	0.86	0.87 (0.66, 1.14)
	Per 0.1 ng/mL		0.0	0.0 (0.0, 0.0)	0.99	0.99 (0.96, 1.01)
**PFNA**	Q1	40 (39-41)	Ref.	Ref.	Ref.	Ref.
	Q2	40 (39-41)	0.1	0.1 (-0.2, 0.3)	0.83	0.84 (0.44, 1.60)
	Q3	40 (39-41)	-0.1	-0.1 (-0.4, 0.1)	0.86	0.87 (0.46, 1.65)
	Q4	40 (39-41)	0.0	0.0 (-0.2, 0.3)	1.17	1.18 (0.65, 2.16)
	Per IQR (0.4 ng/mL)		0.0	0.0 (0.0, 0.1)	0.98	0.99 (0.81, 1.20)
	Per 0.1 ng/mL		0.0	0.0 (0.0, 0.0)	0.99	1.00 (0.94, 1.05)
**PFDA**	Q1	40 (39-41)	Ref.	Ref.	Ref.	Ref.
	Q2	40 (39-41)	0.1	0.1 (-0.2, 0.3)	0.81	0.83 (0.44, 1.58)
	Q3	40 (39-41)	0.1	0.1 (-0.2, 0.3)	0.82	0.82 (0.43, 1.57)
	Q4	40 (39-41)	0.1	0.1 (-0.2, 0.3)	1.06	1.06 (0.58, 1.92)
	Per IQR (0.2 ng/mL)		0.0	0.0 (0.0, 0.1)	0.98	0.98 (0.81, 1.18)
	Per 0.1 ng/mL		0.0	0.0 (0.0, 0.1)	0.99	0.99 (0.89, 1.10)
**PFUnA**	Q1	40 (39-41)	Ref.	Ref.	Ref.	Ref.
	Q2	40 (39-41)	0.0	0.0 (-0.2, 0.3)	0.92	0.91 (0.50, 1.67)
	Q3	40 (39-41)	0.1	0.1 (-0.2, 0.3)	0.85	0.84 (0.44, 1.59)
	Q4	40 (39-41)	0.1	0.1 (-0.1, 0.4)	0.85	0.81 (0.43, 1.51)
	Per IQR (0.2 ng/mL)		0.0	0.0 (-0.1, 0.1)	0.95	0.93 (0.75, 1.16)
	Per 0.1 ng/mL		0.0	0.0 (0.0, 0.1)	0.98	0.97 (0.89, 1.06)

Abbreviations: Quartile (Q), interquartile range (IQR), odds ratio (OR). See PFAA abbreviations in Table 1 in the main document. Preterm birth was defined at births before 37 gestational weeks and 0 days.

References

Textor J, Hardt J, Knüppel S. 2011. DAGitty: a graphical tool for analyzing causal diagrams. Epidemiology 22:745; doi:10.1097/EDE.0b013e318225c2be.
